# AMD and the alternative complement pathway: genetics and functional implications

**DOI:** 10.1186/s40246-016-0079-x

**Published:** 2016-06-21

**Authors:** Perciliz L. Tan, Catherine Bowes Rickman, Nicholas Katsanis

**Affiliations:** Center for Human Disease Modeling, Duke University Medical Center, Durham, NC 27710 USA; Department of Cell Biology, Duke University Medical Center, Durham, NC 27710 USA; Department of Ophthalmology, Duke Eye Center, Duke University, Durham, NC 27710 USA

## Abstract

Age-related macular degeneration (AMD) is an ocular neurodegenerative disorder and is the leading cause of legal blindness in Western societies, with a prevalence of up to 8 % over the age of 60, which continues to increase with age. AMD is characterized by the progressive breakdown of the macula (the central region of the retina), resulting in the loss of central vision including visual acuity. While its molecular etiology remains unclear, advances in genetics and genomics have illuminated the genetic architecture of the disease and have generated attractive pathomechanistic hypotheses. Here, we review the genetic architecture of AMD, considering the contribution of both common and rare alleles to susceptibility, and we explore the possible mechanistic links between photoreceptor degeneration and the alternative complement pathway, a cascade that has emerged as the most potent genetic driver of this disorder.

## Background

Age-related macular degeneration is the third leading cause of vision loss worldwide. It is a late-onset disease with a complex etiology. Major risk factors contributing to susceptibility include age, family history, and smoking [1–3]. The earliest clinical manifestations of age-related macular degeneration (AMD) are focal deposits of debris, termed drusen, which are also considered to be a normal part of aging, present almost ubiquitously in the eyes of healthy individuals over the age of 50. Progression into the spectrum of pathological consequences begins with excessive accumulation of drusen in the central retina during the early/intermediate stages of AMD, followed by localized inflammation, and ultimately neurodegeneration in the macula that characterizes advanced stages of AMD.

Combining epidemiological and genetic approaches has enabled the identification of environmental and genetic contributors to AMD, both of which have tracked with technological advances in conceptual and practical statistical and genomic tools. Linkage of an AMD locus to 1q32 [4, 5], and the genome-wide association at the complement factor H (*CFH*) locus [6] led to the identification of the first common genome-wide significant risk variant, Y402H, that has upon sequencing in ethnic stratified cohorts revealed differential frequencies ranging from 5 % in East Asian populations to 35 % in European populations [7, 8]. This discovery propagated numerous genetics and genomic studies that have contributed to our understanding of the pathomechanisms contributing to AMD. Notably, the subsequent association of common and rare alleles at or near several additional complement genes (*CFH*, *C2/CFB*, *C3*, *CFI*, and *C9)* has had a significant impact of the formation of pathomechanistic hypotheses, with the cumulative evidence both from human genetics but also from histopathological studies highlighting a major role of the alternative complement pathway as a driver of AMD [9–21]. Here, we synthesize genetic evidence from rare- and common-allele studies in AMD, and we discuss the emergent picture of the genetic architecture of this atypical complex trait. Moreover, benefiting from the discovery of likely potent coding mutations in genes encoding complement components, we explore how these mutations might impede specific functions and discuss the potential contribution of aspects of this pathway to AMD pathogenesis.

## AMD histopathology

**Fig. 1:**
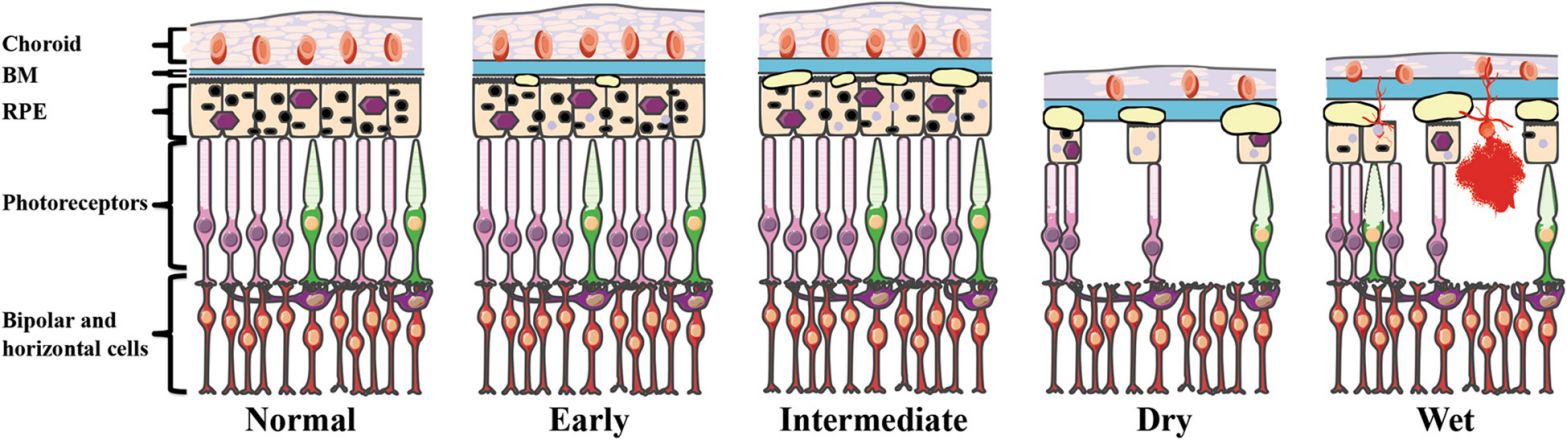
Illustration of the anatomical retinal pathology associated with the various AMD subtypes. Diagram of the outer layers of the human central retina in normal and in AMD. As the disease progresses, Bruch’s membrane (BM) increases in thickness. Early AMD is associated with small drusen and retinal pigmented epithelium (RPE) pigment abnormalities. As the disease progresses to the intermediate form, additional drusen are observed. In the two late forms of AMD (Dry and Wet), there is extensive drusen and photoreceptor cell death, with atrophy of the RPE and choroid in the Dry form and choroidal neovascularization (CNV), hemorrhaging, and RPE detachment in the Wet form. In all forms, the underlying circuitry including the horizontal and bipolar cells remains intact initially. This figure was prepared using Servier Medical Art (http://www.servier.com/Powerpoint-image-bank)

The retina is composed of five major layers: the neurosensory retina; the retinal pigment epithelium (RPE); Bruch’s membrane (BM); the choriocapillaris; and the choroid (Fig. 1). The sensory cells of the neural retina are the photoreceptors (rods and cones), which through phototransduction convert light into electrical signals. Adjacent to the photoreceptors is the RPE, a part of the blood-ocular barrier and has numerous functions, including photoreceptor outer segment phagocytosis; regulation of the transportation nutrients; and cytokine secretion [22]. Lining the basal side of the RPE is BM, which is a pentalaminar extracellular matrix (ECM) consisting of elastin semipermeable barrier between inner and outer collagenous zones that provides connective tissue-based structural support and transport of waste from photoreceptors and RPE to the choroid and nutrients from the choroid to the RPE. The outermost layer of BM is composed of the basement membrane of the choriocapillaris, a fenestrated capillary bed that together with the choroid, a highly vascularized, pigmented tissue, are responsible for supplying the high metabolic demands of oxygen and nutrients to the outer retina [23].

During the natural process of aging, the human eye undergoes physiological changes that include changes in the distribution of photoreceptors (30 % loss of rod photoreceptors [24]); thickening of BM [25]; and accumulation of sub-RPE debris, including drusen [22, 26–29]. Drusen are composed of esterified cholesterol, phospholipids, lipofuscin, inflammatory components (e.g., complement), and other intra- and extraocular degenerative materials [30, 31]. They are classified according to their appearance, with inert, “hard” drusen being small with well-demarcated borders in contrast to the generally pathogenic “soft” drusen that lack distinct borders and can range in size from <63 to >124 μm in diameter, the larger diameters coinciding with disease progression [32].

The clinical pathology of AMD has been described extensively [3, 33]. It involves the progressive degeneration of the macula, a small, pigmented area at the center of the retina that has the densest concentration of photoreceptors and is responsible for visual acuity. Damage to the macula results in loss of central vision. Initial indications of AMD are seen as focal hyperpigmentation of the RPE and accumulation of sub-RPE deposits, including drusen, both between BM and the RPE and within the RPE itself [34, 35].

Even though the degeneration of rods and cones does define the end-stage of the disorder, AMD is not a primary disease of the photoreceptor; most of the candidate processes, biochemical pathways, and molecules exert their effects primarily on the RPE-choroid complex [36].

### AMD subtypes

The anatomical histopathology and clinical progression contribute to the clinical definition of four major AMD subtypes as categorized by the Age-Related Eye Disease Study (AREDS) severity scale grading system [37]: 1. Early AMD; 2. Intermediate AMD; 3. Advanced non-neovascular (“Dry” or geographic atrophy) AMD; and 4. Advanced neovascular (“Wet” or exudative) AMD [32, 38, 39]. Early AMD features few small (<63 μm) or medium-sized drusen (63–124 μm) and pigmentary abnormalities in the RPE, resulting in either mild visual impairment (blurred vision or decreased contrast sensitivity) or can be asymptomatic. The progression from early to intermediate AMD is hallmarked by the appearance of at least one large druse (>125 μm), along with numerous medium-size drusen. This pathology can progress to one of the two advanced forms of AMD: Dry AMD (non-neovascular), characterized by presence of drusen and atrophy of the RPE and choroid; or Wet AMD (neovascular), defined by newly formed vessels (choroidal neovascular membranes (CNV)) and RPE detachment. While occurring gradually in the Dry form versus suddenly/profoundly in the Wet form, the end result of both of these processes leads to photoreceptor cell death and vision loss (Fig. 1, Table 1).

**Table 1:** Characteristics of AMD Clinical Subtypes (based on AREDS)

		AMD Subtype			
		Early	Intermediate	Advanced Dry/Geographic Atrophy	Advanced Neovascular/Exudative/Wet
Clinical Features	Drusen	Few small (<63 μm) or medium sized (63–124 μm)	Numerous medium (63–124 μm) + at least 1 large (>125 μm) sized	Extensive medium (63–124 μm) + large (>125 μm) sized	Extensive medium (63–124 μm) + large (>125 μm) sized
	Bruch’s membrane	Thickening	Thickening	Thickening	Thickening
	Retinal pigment epithelium	Pigmentary abnormalities	Pigmentary abnormalities; atrophy excluding fovea	Atrophy including fovea/macula	Detachment
	Choroid/choriocapillaris	Decreased vascular density	Decreased vascular density	Atrophy	Choroidal neovascularization; hemorrhage; leak fluid
	Neural retina	None	Photoreceptor thinning above drusen	Photoreceptor cell death	Photoreceptor detachment and cell death
	Impairment/outcome	Mild visual impairment (blurred vision or decreased contrast sensitivity) or asymptomatic	Mild visual impairment (blurred spots in central vision or decreased contrast sensitivity) or asymptomatic	Blurred central vision; gradual vision loss	Sudden/profound vision loss

## Genetics of AMD: common variants versus rare variants

AMD exemplifies complex disorders: natural history and epidemiological studies have highlighted a prominent environmental role in disease risk with factors such as smoking resulting in a relative risk (RR) of >2, diet and obesity (RR > 2), nutritional supplements (odds ratio (OR) ~0.6) all contributing to pathogenicity [40]. In addition, family- and population-based studies have also highlighted that AMD carries a significant genetic burden, demonstrating >45 % concordance found in monozygotic and dizygotic twins as well as a recurrence-risk ratio between three to six times greater in siblings than in the general population [41–46].

In many ways, the dissection of the genetic basis of AMD has been an early beneficiary of advances in genetic and genomic technologies, wherein the development of both analytic platforms (genotyping, sequencing) as well as statistical methods, have found early application in this field.

Before our ability to query the genome in toto, complex trait studies focused on more traditional tools such as linkage and candidate gene association studies. Utilizing the latter, AMD was shown to be associated with two genes, *ABCA4* (ATP-binding cassette subfamily A member 4) and *APOE* (apolipoprotein E), both of which only confer a minor risk contribution to AMD. *ABCA4* is a causal gene for Stargardt disease, a retinopathy whose clinical symptoms overlap with AMD [47, 48]; *APOE* is a gene associated with Alzheimer’s disease (AD), a neurological degenerative disorder, whose pathology overlaps with AMD [49]. Nonetheless, and despite this initial discovery, the candidate gene approach was largely unsuccessful in identifying genetic contributors to AMD. This was due in part to limited cohort sizes, imperfect statistical methodologies, and unclear pathomechanisms hindering the ability to define candidate genes and in part due to our lack of appreciation of the extensive rare and common variation carried in human genomes [50–52].

In the era following the completion of the human genome project and the International HapMap Project, traditional family-based studies retained utility, as exemplified by the family-based linkage mapping of an AMD locus on 1q25-q31 and on 10q26 [4, 53]. However, transformative genetic progress in AMD was driven by the common-allele hypothesis explored through genome-wide association studies (GWAS) [6, 54]. Remarkably, modest GWAS analysis on 96 cases and 50 controls yielded a genome-wide significant signal (*P* < 10^−7^) on 1q32 of a common single nucleotide polymorphism (SNP) on a gene encoding *CFH* that revealed a polymorphism in linkage disequilibrium, Y402H, that has proven to be a common, predisposing allele that confers a two- to sevenfold increased risk for developing AMD [6, 9, 55, 56].

Soon after the identification of the *CFH* locus, another modestly sized GWAS performed on 96 cases and 130 controls yielded a genome-wide significant signal on 10q26, another primary locus for AMD that contains the candidate genes *ARMS2*/*HTRA1* (age-related maculopathy susceptibility 2/HtrA serine peptidase 1) coding for a protein of unknown function and a serine protease, respectively [54, 57]. Thereafter, additional GWAS studies with progressively increasing cohort size led to the identification of more associated loci but generally resulted in diminishing returns with many of the loci having a minor contribution to AMD (Table 2).

**Table 2:** Past genome-wide association studies of AMD

Discovered gene/locus	Method	# Cases/Controls	OR	Reference
*CFH*	Affymetrix GeneChip Mapping 100K Set of microarrays	96/50	4.6–7.4	[6]
*HTRA1/LOC387715*	Affymetrix GeneChip Mapping 100K Set of microarrays	99/131	1.66–11.14	[54]
*LIPC*	Affymetrix SNP 6.0 GeneChip and Sequenom	979/1709	0.82	[124]
*CETP*	Affymetrix SNP 6.0 GeneChip and Sequenom	979/1709	1.15	[124]
*TIMP3*	Illumina Human370 Bead Chips and Illumina Infinium II assay	2157/1150	0.63	[125]
*SKIV2L*	Affymetrix SNP 5.0 GeneChip	1896/1866	0.54	[126]
*MYRIP*	Affymetrix SNP 5.0 GeneChip	1896/1866	0.86	[126]
*TNFRSF10A/LOC389641*	Illumina Human610-Quad BeadChip and Illumina HumanHap550v3 BeadChip	1536/18894	0.73	[127]
*FRK/COL10A1*	Affymetrix SNP 6.0 GeneChip and Illumina HumanCNV370v1 Bead Array	2594/4134	0.87	[128]
*VEFGA*	Affymetrix SNP 6.0 GeneChip and Illumina HumanCNV370v1 Bead Array	2594/4134	1.15	[128]
*COL8A1/FILIP1L*	Meta-analysis of GWAS	>17000/>60000	1.23	[129]
*APOE*	Meta-analysis of GWAS	>17000/>60000		[129]
*IER3/DDR1*	Meta-analysis of GWAS	>17000/>60000	1.16	[129]
*SLC16A8*	Meta-analysis of GWAS	>17000/>60000	1.15	[129]
*TGFBR1*	Meta-analysis of GWAS	>17000/>60000	1.13	[129]
*RAD51B*	Meta-analysis of GWAS	>17000/>60000	1.11	[129]
*ADAMTS9/MIR548A2*	Meta-analysis of GWAS	>17000/>60000	1.1	[129]
*B3GALTL*	Meta-analysis of GWAS	>17000/>60000	1.1	[129]

Meta-analysis genotyping method/platforms include Illumina HumanHap300, Human610-Quad, HumanHapCNV370, or HumanCNV370v1 BeadChips, Affymetrix 250K Nspl, Illumina Human670-QuadCustom chip, Illumina 660-Quadv1A, Illumina 610-Quad, Illumina Infinium HumanHap300K, HumanHap550v1, or HumanHap550v3 BeadChip, Illumina Infinium II HumanHap550, Affymetrix GeneChip Human Mapping 250k Styl Array, Affymetrix 6.0 or 1M

The associated loci discovered also germinated targeted sequencing strategies with a goal of identifying rare variants that might (a) provide direct causal evidence for the gene(s) in associated regions; (b) improve our measure of the overall risk of such genes to AMD pathogenesis; and (c) inform the direction of effect. Targeted high-throughput sequencing in combination with genotyping of the *CFH* locus led to the identification of a highly penetrant, rare variant R1210C which was shown to be associated with advanced AMD and to potentially result in disease onset 6 years earlier [58]. Similarly, in a smaller discovery cohort of 84 unrelated AMD patients, another penetrant, rare missense mutation in complement factor I (*CFI*), was unveiled, encoding G119R. This allele confers high risk to AMD (OR = 22.2), with patients with the heterozygous mutation having lower FI serum concentration [19].

With a goal of identifying elusive rare variants, a larger targeted exon sequencing study was undertaken in 2013, in which all exons of the 681 genes mapping at or close to all the AMD reported loci were sequenced in 1676 cases, 745 controls, and 36 siblings with discordant disease status [59]. This study combined sequencing-based genotypes, with the exome chip genome-wide genotyping data, with SNP genotyping to uncover K155Q (OR = 2.8) in the complement component C3 gene (*C3)* and P167S (OR = 2.2) in *C9* as contributors to AMD disease pathophysiology. Importantly, the study also showed an overall increase in the burden of rare variants in cases compared to controls. Much of that signal originated from specific genes, such as *CFI* (7.8 % in cases compared to 2.3 % in controls). However, the data suggested that there were variants in additional genes that were likely involved but could not be powered sufficiently to show association for specific alleles [59]. More recent studies have involved a targeted capture enriched for complement components that led to discovery of enrichment of rare variants in *CFH* in AMD patients [60] as well as the largest AMD GWAS study to date that examined >12 million variants in 16,144 patients and 17,832 controls [61]. This study leads to the identification of 52 common and rare variants across 34 loci, 16 of which reached genome-wide significance for the first time [61]. Taken together, these studies highlight two key points. First, they showed that, for AMD, a blend of rare and common alleles can contribute substantially to the disease burden and, as accepted by evolutionary theory, coding alleles of major functional effect can have a much larger contribution to susceptibility. Second, however, was the more sobering observation that AMD is quite different from most other common traits studied to date. A few exceptions notwithstanding, the genetic architecture of AMD has intimated the presence of a few genes in which common, modestly penetrant alleles account for a significant fraction of the genetic burden, with rare coding variants in the same genes providing both causal evidence and adding further to the population burden [6, 58, 62, 63]. When juxtaposed with the >1000 GWAS executed for a variety of traits, this landscape has, for the most part, been confined to this disorder. The reason for this is unclear. These observations might underscore a “winner’s curse” of the first major complex trait’s GWAS successes. More importantly, they raise the possibility that the biochemical underpinnings of AMD might be fundamentally different from other complex traits and that understanding the reasons for such differences might inform our approach to both genetic discovery and therapeutic design.

Also contrasting other GWAS approaches in other diseases, a significant fraction of the susceptibility signal in AMD has mapped to, or near, genes encoding components of the complement cascade, although this is not the sole pathway implicated. Overall, the genes and alleles thought to confer significant susceptibility to AMD pathology can be clustered broadly into five major pathways (Table 3): (a) the inflammation and immune response, (b) lipid metabolism and transport, (c) extracellular matrix and cell adhesion, (d) angiogenesis, and (e) cellular stress responses. Among these, risk assessment analysis for either common or rare alleles has highlighted in genes encoding complement pathway components. The common variants near six complement genes, *CFH*, *C2/CFB*, *C3*, *CFI*, and *C9* together, account for almost 60 % of the AMD genetic risk [36]. Notably, for the rare alleles found in sufficient recurrent rates to empower meaningful studies, the individual risk to AMD is sharply higher and appears as if they are almost Mendelian, as observed in the case of *CFI* [19].

**Table 3:** Genes associated with AMD that cluster into five major pathways

Inflammation and immune response				Cell stress response			
C2	Complement component 2	HLA-C	Major histocompatibility complex, class I, C	ABCA4	ATP-binding cassette subfamily A member 4	HTRA1	HtrA serine peptidase 1
C3	Complement component 3	IL8	Interleukin B	ACE	Angiotensin I converting enzyme 1	RORA	RAR-related orphan receptor alpha
CFB	Complement factor B	MMP9	Matrix metallopeptidase 9	APOE	Apoliporotein E	SOD2	Superoxide dismutase 2, mitochondrial
CFH	Complement factor H	PLEKHA1	Pleckstrin homology domain containing, family A member 1	ARMS2	Age-related maculopathy susceptibility 2	TF	Transferrin
CFD	Complement factor D	RORA	RAR-related orphan receptor alpha	CST3	Cystatin C	TLR3	Toll-like receptor 3
CFHR1-5	Complement factor H-related 1-5	SERPING1	Serpin peptidase inhibitor, clade G, member 1	CX3CR1	Chemokine receptor 1	TLR4	Toll-like receptor 4
CFI	Complement factor I	TLR3	Toll-like receptor 3	CYP24A1	Cytochrome P450, family 24, subfamily A peptide 1	VLDLR	Very low-density lipoprotein receptor
C9	Complement component 9	TLR4	Toll-like receptor 4	GSTM1	Glutathione S-transferase mu 1	TNFRSF10A/LOC389641	Tumor necrosis factor receptor superfamily, member 10a
CX3CR1	Chemokine receptor 1	VLDLR	Very low-density lipoprotein receptor	GSTP1	Glutathione S-transferase pi 1	IER3	Immediate early response 3
F13B	Coagulation factor XIII, B polypeptide	VTN	Vitronectin	GSTT1	Glutathione S-transferase tau 1	TGFBR1	Transforming growth factor, beta receptor 1
Lipid metabolism and transport				Extracellular matrix and cell adhesion			
ABCA1	ATP-binding cassette, subfamily A, member 1	FADS1-3	Fatty acid desaturases 1-3	ACE	Angiotensin 1 converting enzyme 1	ROBO1	Roundabout, axon guidance receptor, homolog 1
ABCA4	ATP-binding cassette, subfamily A, member 4	LIPC	Hepatic lipase	ARMS2	Age-related maculopathy susceptibility 2	TIMP3	Tissue inhibitor of metalloproteinase 3
APOE	Apolipoprotein E	LPL	Lipoprotein lipases	ADAMTS9	ADAM metallopeptidase with trhombospondin type 1 motif, 9	MMP19	Matrix metallopeptidase 19
CETP	Cholesteryl ester transfer protein, plasma	LRP6	Low-density lipoprotein receptor-related protein 6	COL8A1	Collagen, type VIII, alpha 1	PCOLCE	Procollagen c-endopeptidase enhancer
CFHR1-5	Complement factor H-related 1-5	RORA	RAR-related orphan receptor alpha	COL10A1	Collagen, type X, alpha 1	VTN	Vitronectin
CYP24A1	Cytochrome P450, family 24, subfamily A peptide 1	VLDLR	Very low-density lipoprotein receptor	CST3	Cystatin C	ABCA7	ATP-binding cassette, cubfamily A, member 7
ELOVL4	ELVL fatty acid elongase 4	PLTP	Phospholipid transfer protein	CX3CR1	Chemokine receptor 1	ACTG1	Actin gamma 1
Angiogenesis				F13B	Coagulation factor XIII, B polypeptide	BCAR1	Breast cancer anti-estrogen resistance 1
ACE	Angiotensin I converting enzyme 1	LRP6	Low-density lipoprotein receptor-related protein 6	FBLN5	Fibulin 5	COL4A4	Collagen, type IV, alpha 4
COL10A1	Collagen, type X, alpha 1	MMP9	Matrix metallopeptidase 9	HMCN1	Hemicentin	ITGA7	Integrin, alpha 7
COL8A1	Collagen, type VIII, alpha 1	RORA	RAR-related orphan receptor alpha	HTRA1	HtrA serine peptidase 1	MYL2	Myosin, light chain 2, regulatory, cardiac, slow
CST3	Cystatin C	SERPINF1	Serpin peptidase inhibitor, clade F	MMP9	Matrix metallopeptidase 1		
FBLN5	Fibulin 5	TIMP3	Tissue inhibitor of metalloproteinase 3				
GDF6	Growth differentiation factor 6	VEGFA	Vascular endothelial growth factor A				
HTRA1	HtrA serine peptidase 1	VLDLR	Very low-density lipoprotein receptor				
IL8	Interleukin 8						

## A pathogenic route to AMD: alternative complement pathway

One of the leading candidates for predisposition to AMD is the inflammatory pathogenesis theory, which hypothesizes dysregulation of the immune response, specifically complement system [6, 9, 12, 14, 15, 20, 55, 56, 64–68]. Reports dating back to 1875 have hypothesized that macular lesions were due to inflammation and that disciform degeneration were associated with choroidal inflammation [69]. Since then multiple components of the complement pathway have been linked to AMD and its pathological consequences [15, 16, 30, 64, 70, 71]. The complement system is a specialized part of innate immunity that can respond to antigen-antibody complexes (classical pathway) or bacterial mannose groups (lectin pathway) and can also be active in a low-level continuous state (alternative pathway (AP)), to allow for an immediate amplified response [72]. All complement pathways culminate in the creation of the membrane attack complex (MAC) for cell lysis and organismal defense.

AMD is not the first human genetic disorder associated with AP complement dysfunction. A number of disorders are thought to be the result of excessive AP activation, including membranoproliferative glomerulonephritis type II (MPGN type II); atypical hemolytic uremic syndrome (aHUS); and paroxysmal nocturnal hemoglobinuria (PNH, a rare form of hemolytic anemia). Of note, MPGN type II, which is characterized by renal disease and low serum C3 levels, is also associated with complete FH deficiency, arguing for a mechanistic similarity between MPGN and AMD [55, 73]. Similarly, aHUS has been associated with low FH levels in addition to low C3 levels and has mutations in several AP components, however, at a unique haplotype compared to AMD and MPGN [73]. PNH is caused by mutations in phosphatidylinositol glycan-complementation class A (PIGA) that is essential in the establishment of glycophosphatidylinositol anchors. Two known inhibitors of AP activation are glycolipid-anchored proteins that are required for the regulation at the C3 convertase step and MAC assembly [74].

Dysregulation of the AP specifically is currently thought to underlie AMD. Several lines of supporting evidence include (1) the presence of complement components in the choriocapillaris and the retina, especially in drusen [14–16, 75]; (2) increased MAC in the choriocapillaris of AMD patients [18]; and (3) the genetic association of *C2*, *C3*, *CFB*, *CFI*, and the regulator of complement activation (RCA) gene cluster on 1q32 (which includes *CFH* and *CFHR1-5*) with AMD [9–13, 17, 19–21]. However, the effects of variation within the components of the AP on AMD pathogenicity is largely unknown.

## AMD is associated with activators of alternative pathway components

**Fig. 2:**
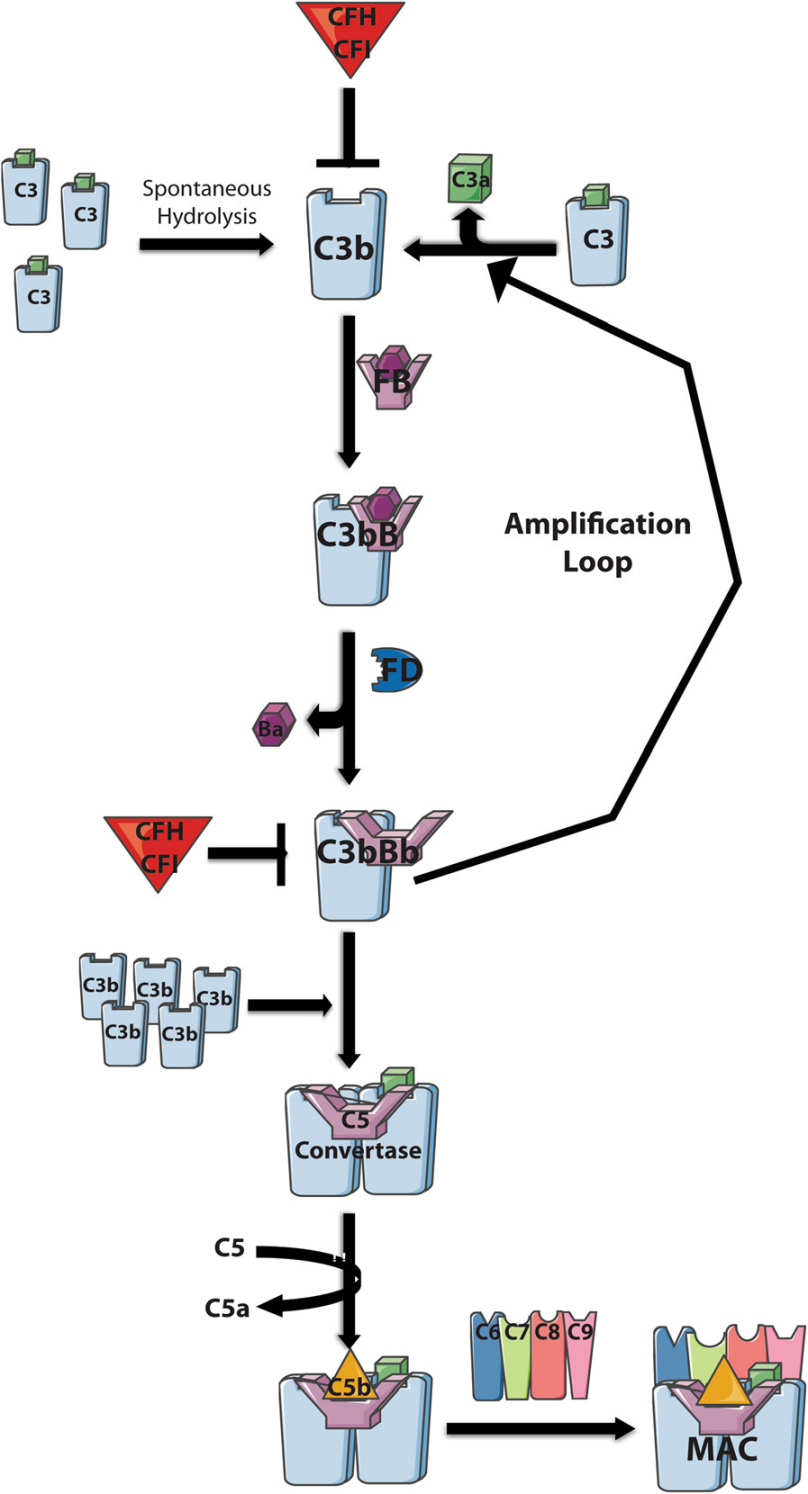
The alternative complement pathway and the formation of the C3 convertase. In the AP, the generation of C3b can occur by either spontaneous hydrolysis of C3 (“tick-over” allowing for continuous low-level activation) or by plasma proteolytic cleavage all allowing for immediate C3b deposition. C3b forms the C3 convertase upon binding to FB and cleavage by FD resulting in an amplification loop producing additional C3b to stimulate a large immune response. C3b additionally binds to the C3 convertase leading to the formation of the C5 convertase initiating the terminal pathway and the establishment of the MAC. This figure was prepared using Servier Medical Art (http://www.servier.com/Powerpoint-image-bank)

Low levels of constitutive complement activation via the AP allows for immediate immune response. It involves the central molecule of all three arms of the complement pathway, C3. In the AP, C3, is activated in two ways (Fig. 2): (1) it is either cleaved by convertases/plasma proteases to generate C3b or (2) a small portion is hydrolyzed spontaneously to C3_H2O_ establishing a continuous “tick-over” ready for immediate C3b deposition on pathogens for target opsonization [76–79]. Factor B (FB) binds to C3_H2O_ or C3b and the complex is cleaved by the plasma protease factor D (FD) forming the essential C3 convertase (C3bBb or C3_H2O_Bb), leading to an amplification loop that cleaves and assembles C3 to C3b to C3bBb continuously. The accumulation of C3b leads to C3b binding to C3bBb, thereby creating a new enzyme, the C5 convertase (C3bBbC3b) that cleaves C5 to C5a (an anaphylatoxin and chemoattractant) and C5b, a component of the lytic pore which combines with C6–C9 to form the membrane attack complex (MAC) resulting in cell lysis [72].

The AP has the unique characteristic of being able to be activated spontaneously, allowing for an immediate immune response, which must be tightly regulated to prevent excessive activation. Intrinsically, the AP has two major negative regulators encoded by *CFH* and *CFI. CFH* is the soluble inhibitor of the complement cascade and encodes a secreted glycoprotein, FH that acts as cofactor for FI mediated cleavage of C3b as well as accelerating decay of the C3bBb convertase.

Many cells also express membrane-associated AP regulators that hinder the C3 amplification loop and/or prevent the deposition/accumulation of C3b on self-tissue. These membrane-associated proteins include membrane cofactor protein (MCP), decay-accelerating factor (DAF), and complement receptor 1 (CR1) all of which are encoded by genes located within the *RCA* gene cluster on 1q32 [80]. MCP and CR1 have cofactor activity for CFI*,* while CR1 along with DAF have decay acceleration activity. Differential expression of *MCP* has been linked to AMD and upon the addition of an environmental stimulus such as smoking, a risk factor of AMD, both MCP and DAF are down regulated [81, 82]. Both activating and inhibiting components have been reported to harbor variants that potentially have functional impacts on the AP (Table 4), with an increase in overall mutational burden being reported in *CFH* and *CFI* in AMD patients [60, 83]. These perturbations of the components themselves or of their ability to interact with other AP components typically result in uncontrolled AP activation.

**Table 4:** AP complement variants

Gene	Variant	Position	OR	Common/Rare	Domain	Variant domain effect	Effect on AP	Ref
*C3*	R102G	19:6718387	1.7–2.6	Common	Macroglobulin 1	Decreased binding efficiency of C3b to its inhibitor CFH decreasing CFH’s cofactor activity	Increased AP	[20, 65]
*C3*	P314L	19:6713262	1.5	Common	Macroglobulin 3	Decreased binding efficiency of C3b to its inhibitor CFH decreasing CFH’s cofactor activity	Increased AP	[65]
*C3*	K155Q	19:6718146	2.8–3.8	Rare	Macroglobulin 2	Reduces binding to FH and C3b proteolytic cleavage by CFI	Increase C3 convertase production	[59, 65]
*CFB*	R32Q	6:31914180	0.32	Common	Ba	Reduced affinity for C3b and reduced hemolytic activity	Decrease formation of C3 convertase; reduced AP	[130]
*CFB*	L9H	6:31914024	0.37	Common	Signal peptide	Affects secretion of CFB	Decrease formation of C3 convertase; reduced AP	[12]
*CFH*	Y402H	1:196659291	2.45–5.57	Common	Complement control protein 7	Affects binding to GAG heparin sulfate, sialic acid, and C-reactive protein	Reduces ability of CFH to degrade C3: increased AP	[131]
*CFH*	R1210C	1:196716375	23.11	Rare	Complement control protein 20	Defective binding to C3b, C3d, and heparin	Impaired CFH attachment to host surfaces; reduces AP regulation	[58]
*CFH*	R53C	1:196642206		Rare	Complement control protein 1	Reduced accelerating activity for AP C3 convertase	Altered CFH-mediated cofactor activity or decay-accelerating activity; increased AP	[63]
*CFH*	D90G	1:196643011		Rare	Complement control protein 2	Alters CFI cofactor activity	Decreased cofactor-mediated inactivation; increased AP	[63]
*CFH*	I62V	1:196642233	1.95–2.79	Common	Complement control protein 2	Binds more efficiently to C3b	Enhanced cofactor activity and increased formation of iC3b; reduced AP	[63]
*CFH*	N1050Y	1:196712596	0.4	Common	Complement control protein 18	Possibly affects GAG and sialic acid binding	Increased AP	[62]
*CFHR1/3*	CFHR1/3del		0.29	Common	n/a	n/a	Reduces cofactor activity for CFI and inhibits C5 convertase; reduced AP	[13] [132–135]
*CFI*	G119R	4:110685820	22.2	Rare	Scavenger receptor cysteine-rich	Perturbs interdomain packing and stability of CFI	Diminished ability to degrade C3b; increased formation of C5 convertase; increased AP	[19]
*CFI*	G188A	4:110682768		Rare	Scavenger receptor cysteine-rich	Perturbs interdomain packing and stability of CFI	Diminished ability to degrade C3b; increased formation of C5 convertase; increased AP	[19]
*C9*	P167S	5:39331894	2.2	Rare	Membrane attack complex/perforin	Alters oligomerization and possibly inhibits C9's lytic activity	Affects binding with CD59; alters pore formation	[59]

## Pathogenic outcomes of the AP

Advanced age is the only risk factor common to all AMD patients. During the aging process, the physiological changes include redistribution of the photoreceptors, thickening of BM, and accumulation of debris in the eye in conjunction with environmental factors and genetic variation contribute to cascading dysfunction of physiological pathways such as lipid transport, angiogenesis, stress response, and ECM remodeling [24–26]. In turn, disruption of each of these pathways can lead to an inflammatory and an AP immune response ultimately culminating in cell death [72]. Each of these pathways has been hypothesized to alter components of the retina [36, 84] affecting the interdependence of the photoreceptors, RPE, BM, and choriocapillaris making it challenging to understand the model for AMD pathogenesis.

During the early stages of AMD, sub-RPE deposit formation (drusen and soft basal linear deposits) between the RPE and BM occurs and is also thought to be the main site of immune complex formation in AMD. While containing over 40 % lipids, other components of sub-RPE deposits include TIMP3 (TIMP metallopeptidase inhibitor 3), which plays a role in ECM maintenance and remodeling [85, 86]; amyloid beta (Aβ) that is produced either systemically or from the RPE and is proangiogenic and a known activator of complement [87]; apolipoproteins, which are also generated systemically and by the RPE [88]); and CFH and other complement components [30].

Lipid accumulation, similar to that seen in atherosclerosis, can increase choroidal vessel resistance preventing the choriocapillaris from properly clearing additional lipoproteins from the RPE and BM [89]. The accumulation of lipoproteins along with Aβ lead to the formation of a lipid “wall” external to the RPE [90, 91] that is the precursor to basal linear deposits that forms between the RPE basement membrane and the inner collagenous zone of BM [92]. The accumulated, peroxidizable lipoproteins are oxidized contributing to RPE damage [93]. Additionally, one byproduct of lipid peroxidation product is malondialdehyde (MDA), a marker for oxidative stress that contributes to RPE dysfunction [94] and a binding partner to CFH, allowing for an endogenous anti-inflammatory mediated response to this pro-inflammatory byproduct [95]. Similar to MDA, another cholesterol oxidation product having pro-inflammatory effects is 7-ketocholesterol (7KCh), which has been associated with cytotoxicity and has been shown to interact with retinal microglia, possibly promoting choroidal neovascularization [96] [97]. Further, these oxidized low-density lipoproteins can bind C-reactive protein initiating an inflammatory response leading to complement activation [98, 99]. Supplementing evidence has also been reported that complement activation leads to the recruitment of mononuclear phagocytes that contribute to RPE pathology in AMD [100]. All points that support immune system activation as a consequence of pathologic lipid accumulation.

The majority of complement is synthesized primarily by the liver and is delivered through circulation; however, some local tissues are also able to synthesize complement components, specifically the RPE and choroid [55]. Upon factors such as aging, oxidative stress (including cigarette smoke which that has been reported in vitro to activate C3 [101]), and lipid accumulation, increased AP activation in the RPE, which is thought to be the site of primary dysfunction in AMD, is primed for genetic predispositions to onset disease progression [14, 75, 101]. Upon AP activation, initiation of the terminal pathway ensues, forming MAC in BM and the choriocapillaris contributing to compromised function of the tight interaction of the RPE-BM-choroid complex [18].

## Additional clues and emerging thoughts on pathogenic contributions to complex disease

There have been multiple insights implicating complement components in the pathobiology of AMD including genetic associations and accumulation of complement components in sub-RPE deposits of patient retinas [30, 102]. With common variants accounting for 65 % of the heritability of AMD, the search for rare variant contributors has only recently been undertaken through the advances of next generation sequencing [40]. Rare variants of C3, CFH, CFI, and C9 that have thus far been associated with AMD have been shown to play a role in either the complement pathway, to have an impact on the mutational load, or to hold the promise of putative future therapeutic targets [19, 58, 59, 63, 65]. However, a leading limitation in understanding disease outcome has been the lack of comprehension of the functional impact of the genetic contributors themselves. By combining the common variant common disease and rare variant common disease hypotheses [103–105], the idea of mutational burden emerges as a prognostic/diagnostic alternative for AMD, as exemplified by both common and rare variants in AP inhibitors leading to pathogenic dysregulated AP activity (Table 4). The latter highlights the necessity to functionally assess the role of a variant(s) in a gene(s) in order to more explicitly understand AMD pathogenesis. This highlights the need for development of animal models which are currently in development, for example, a transgenic mouse model expressing the human normal (Y402) and AMD-risk associated (Y402H) variants of FH has recently been described [106].

Familial aggregation is observed in most complex disease since there is greater likelihood of sharing disease-predisposing genotypes; however, non-genetic factors can contribute to discordant phenotypes [40]. In an effort to understand disease phenotypic outcomes, most studies have started by trying to identify causal variants. The identification of susceptibility variants is hindered by numerous confounding factors that have to be acknowledged to gain insight into disease mechanism. (1) Excluding a few cases such as AD and AMD, the vast majority of common variants exhibit a modest effect therefore large cohorts are required to power the findings [107, 108]. (2) Rare, disease-causing mutations, while numerous, are rarely observed in the general population [60, 83, 109, 110]. (3) SNP association of a locus does not imply that the causal variant itself is a SNP, as observed in Crohn’s disease where the causal mutations is a deletion upstream of the promoter [111]. (4) Multiple distinct alleles either common, rare, or both, can be present at a single locus [58, 112]. 5) Allele frequencies vary among ethnic groups, as not only observed in AMD but also described in Hirschsprung’s disease in which the causal variant has a minor allele frequency (MAF) ranging from 0.01 to 0.45 depending on the population [113]. (6) Causality due to non-coding variants is difficult to establish, similar to what was observed in coronary artery disease [114–116]. (7) Similar to pathways functioning in multiple processes, variants can also be pleiotropic, with their effect being dependent on the genetic content in which they are identified [117]. 8) Genes with causal variants identified need to be further studied for additional variation that could contribute to disease, as was seen in the case when delving deeper into *CFH* and *CFI* in AMD [19, 58]. (9) Structural variation cannot be ignored, as was observed in the case of Bardet-Biedl Syndrome in which copy number variations lead to disease progression (unpublished data). (10) Environmental factors can modify genetic effects and phenotypic outcomes [2, 101, 118].

## Conclusions

Genetics has aided in the understanding of the complex, multifactorial nature of AMD. However, with modest signals differentiating the Dry versus Wet advanced forms of AMD, genetics has not revealed a strong predictive value on phenotypic severity or progression. Despite the long road of discovery that lies ahead, various lines of evidence linking the AP to AMD disease pathogenesis including (a) clinical phenotypes that associate with deficiencies of AP regulators [119]; (b) linkage of SNPs in AP components to disease risk [11, 120]; and (c) the functional analysis of individual AP components in the establishment of clinical phenotypes observed in both mouse and zebrafish models [74]. With numerous mouse models recapitulating at least 10 distinct human disorders, such as rheumatoid arthritis, traumatic brain injury, and AMD, the effects of AP on development and homeostasis have been established, making it clear that dysregulation/dysfunction of the process of innate immunity plays a contributory role in disease outcome [74]. Predominant functional assays for measuring the impact of variation on the complement pathway have been hemolytic and enzyme immunoassays [59, 121–123]; focus should and has begun to be placed on additional in vivo model systems to further understand the roles of AP disease contributors in a more systemic context [100, 106].

## Abbreviations

7KCh: 7-ketocholesterol
ABCA4: ATP-binding cassette subfamily A member 4
AD: Alzheimer’s disease
aHUS: atypical hemolytic uremic syndrome
AMD: age-related macular degeneration
AP: alternative pathway
APOE: apolipoprotein E
AREDS: Age-Related Eye Disease Study
ARMS2: age-related maculopathy susceptibility 2
Aβ: amyloid beta
BM: Bruch’s membrane
C2: complement component 2
C3: complement component 3
C9: complement component 9
CFB or FB: complement factor B
CFH or FH: complement factor H
CFI or FI: complement factor I
CNV: choroidal neovascular membranes
CR1: complement receptor 1
DAF: decay-accelerating factor
ECM: extracellular matrix
FD: complement factor D
GWAS: genome-wide association studies
HTRA1: HtrA serine peptidase 1
MAC: membrane attack complex
MAF: minor allele frequency
MCP: membrane cofactor protein
MDA: malondialdehyde
MPGN: membranoproliferative glomerulonephritis
OR: odds ratio
PIGA: phosphatidylinositol glycan-complement class A
PNH: paroxysmal nocturnal Hemoglobinuria
RCA: regulator of complement activation
RPE: retinal pigment epithelium
RR: relative risk
SNP: single nucleotide polymorphism
TIMP3: TIMP metallopeptidase inhibitor 3
